# Implementation of case-based collaborative learning in radiology residency: experience report of an international educational experience adapted to the Brazilian context

**DOI:** 10.1186/s12909-026-09781-9

**Published:** 2026-07-08

**Authors:** Steven Montague, Aditi Khokhar, Brandy DeWeese, Jeananne Elkins, Morkel Jacques Otto, Ana Amelia Bartolamei Ramos, Julia Priscila Ferraz Pepes, Rosiane Guetter Mello, Orlando Ortiz, Camila Aparecida Moraes Marques

**Affiliations:** 1 Queen’s University, Kingston, ON Canada; 2 Goryeb Children’s Hospital, Morristown, NJ USA; 3 Virginia Mason Franciscan Health, St. Elizabeth Hospital, 1455 Battersby Avenue, Enumclaw, WA USA; 4 Department of Physical Therapy, Texas A&M University, Texarkana, USA; 5 Intravita International Ltd, Triptree, Essex UK; 6 Faculdades Pequeno Príncipe, Curitiba, Brazil; 7 Jacobi Medical Center, Albert Einstein College of Medicine, New York, USA

**Keywords:** Medical education, Case-based collaborative learning, Active methodologies, Radiology, Clinical teaching

## Abstract

**Background:**

Contemporary medical education increasingly incorporates active learning strategies to promote integration between foundational sciences and clinical practice and to support the development of diagnostic reasoning. Case-Based Collaborative Learning (CBCL) combines principles of team-based learning, problem-based learning, and case-based learning through structured preparation and collaborative discussion of clinical cases. In this context, this experience report describes the adaptation and implementation of a CBCL-based educational activity focused on clinical-radiological reasoning in suspected spinal infections within a radiology residency program in Brazil.

**Experience description:**

The activity was conducted in a radiology residency program at a public hospital in Curitiba, Paraná, Brazil, involving 20 residents from different years of training. Participants received preparatory materials covering anatomy, microbiology, pathophysiology, and imaging guidelines from the American College of Radiology before the face-to-face session. The 90-minute CBCL session was conducted in small groups of four participants and organized around clinical cases involving discitis-osteomyelitis, epidural abscess, and differential radiological diagnoses. Each case followed a structured three-step sequence: individual reflection, small-group discussion, and facilitator-led plenary synthesis. Perceived outcomes: The CBCL structure was feasible to implement and was well accepted by participants. Learners demonstrated active participation and high engagement throughout the activity. Immediate feedback suggested perceived contributions to collaborative learning, conceptual integration, and clinical-radiological reasoning. No formal post-intervention knowledge assessment or objective evaluation of educational effectiveness was performed. Most participants reported positive perceptions regarding engagement and collaborative reasoning.

**Conclusions:**

For future educational research that CBCL may represent a feasible and pedagogically promising strategy for radiology residency education. The approach supported active participation and integration between foundational and clinical knowledge within a collaborative learning environment. Future studies using objective educational outcomes and longitudinal evaluation are needed to further investigate its educational impact.

## Introduction

Recent literature has shown that the CBCL (Case-based Collaborative Learning) promotes engagement, meaningful discussion, vertical integration between basic and clinical content, and progressive development of self-regulated learning. The model described by Besche et al. organizes the experience into three interconnected axes: instructional design, facilitation, and professional transformation, highlighting that the quality of the experience depends on the coherence between prior preparation, appropriate case complexity, teacher mediation, and post-class consolidation [1]. In parallel, implementation studies in undergraduate and residency programs indicate that the method can increase student acceptance, applied knowledge, and satisfaction, including face-to-face and remote contexts [2].

In the field of Radiology, this educational intervention is particularly relevant. Radiological reasoning is not limited to image recognition; it requires coordination between clinical practice, anatomy, pathophysiology, microbiology, test interpretation, and knowledge of relevant guidelines. In the teaching material developed within the scope of Training to Teach in Medicine (T2T-M), the educational curriculum was designed for Emergency Medicine residents with a focus on the American College of Radiology (ACR) appropriateness criteria [3] for suspected spinal infection, articulating basic concepts and clinical decision-making based on progressive cases. The present report describes, in detail, the adaptation and implementation of a CBCL session inspired by an international medical educational experience, with an emphasis on its pedagogical organization, session development, theoretical foundations, and training potential. Given the descriptive purpose of an experience report, the present manuscript focuses on curriculum adaptation, implementation processes, feasibility, and learner perceptions rather than on measuring educational effectiveness.”

## Contextualization of the experience

Within the Training to Teach in Medicine (T2T-M) program at Harvard Medical School, an educational module was originally designed by an international team, targeting emergency medicine residents, and focusing on the American College of Radiology (ACR) Appropriateness Criteria [3] for suspected spinal infection. The objectives were to improve the evaluation and management of patients with neck or back pain and suspected spinal infection, recognizing that diagnosis can be clinically challenging, that diagnostic delays have serious consequences, and that the ACR criteria can support imaging investigation decisions [4].

In the base material for the experience, the educational objective was defined as developing and implementing an integrated curriculum capable of improving the evaluation and management of spinal infections in residents, articulating spinal anatomy, microbiology, pathophysiology, radiological findings, and use of the ACR Appropriateness Criteria. The learning objectives included: relating microbiology and spine anatomy to pathophysiology and imaging findings; evaluating clinical and laboratory data to consider the diagnosis; incorporating ACR criteria into assessment and management; and differentiating infectious spondylodiscitis from mimicking diagnoses [5].

We adapted that module to a Brazilian residency setting in a public hospital in Curitiba, Paraná, Brazil. The participants were 20 radiology residents, including trainees from different years of training, divided into five groups of four members. The activity was conducted within the routine residency program, with participation from residents present at the time of the session. No control group was included, as this study is designed as an educational experience report, focusing on the description of implementation and feasibility rather than comparative analysis. The adaptation involved reformulating the language, reorganizing the educational objectives, and emphasizing the clinical-radiological axis. This process was carried out while preserving the core pedagogical structure of CBCL, including pre-session preparation, readiness assessment, small-group case discussion, and facilitated collective synthesis.

### Pedagogical rationale for the educational intervention

The CBCL was chosen for its ability to combine structure and flexibility. In a randomized controlled trial conducted at Harvard, Krupat et al. described the CBCL as a method that transfers readiness to before class, through multiple-choice questions related to the key concepts of the preparatory material and uses face-to-face time exclusively for case discussion with open-ended questions, small group resolution, and broad consensus building. In this study, although overall performance on the final exam did not differ significantly from PBL (Problem-Based Learning), students with previous below-median performance had better results in the CBCL group, and the methodology was perceived as engaging, engaging and intellectually stimulating [6].

The article by Berkowitz and Chang, which describes the development of a preclinical neurology course using a flipped classroom model with CBCL at Harvard, reinforces the importance of consistency between previous materials readiness questions, and cases discussed in class, indicating an approximate 1:1 ratio between preparation time and classroom time, in addition to the use of applied clinical questions to verify preparation before the session [2].

Besche et al. expand on this understanding by arguing that the success of the session depends on preparatory materials with scaffolding, cases with appropriate complexity, open-ended questions that call for inductive reasoning, and teaching facilitation capable of balancing student autonomy and conceptual convergence [2, 6]. In other contexts, the applicability of CBCL has also been documented. Evidence regarding CBCL in medical education has shown consistent positive perceptions related to engagement, collaborative reasoning, and learner satisfaction across undergraduate and residency settings [1–4, 7]. However, much of the available literature remains limited by single-center experiences, heterogeneous implementation models, and a predominance of subjective educational outcomes. While some studies have demonstrated improvements in knowledge scores [4], others primarily report learner perceptions and acceptability [6–7], highlighting the need for additional context-specific implementation experiences and more robust longitudinal evaluation strategies. In a qualitative study conducted with medical students in physiology, participants reported that the CBCL favored knowledge integration, student centrality, constructive debate, teamwork, and better participation when compared to traditional expository methodologies [1, 7].

## Detailed description of the pedagogical intervention

The activity was structured in three main stages: pre-session, case-based face-to-face session, and evaluation of the experience. This organization coincides with the architecture recommended in the CBCL conceptual materials and with the structure presented in the T2T-M project itself [8].

## Stage 1: pre-session preparation

The pre-session phase was structured according to the flipped classroom model adopted within the CBCL framework. Participants received preparatory materials developed from the original T2T-M educational module and aligned with the learning objectives of the session and the American College of Radiology (ACR) Appropriateness Criteria [3] for suspected spinal infection. These materials included a short video (approximately five minutes) covering three essential conceptual domains, spine anatomy, microbiology, and vertebral infection, as well as two PDF documents: a summary of the ACR criteria and an introductory clinical case for prior study.

To support learner readiness, a brief formative assessment composed of five multiple-choice questions (MCQs) was administered before the face-to-face session. This assessment functioned as a readiness assurance/self-assessment activity rather than a summative evaluation and aimed to promote minimum familiarity with the concepts necessary for meaningful participation in the collaborative discussions [9]. The MCQs were not formally validated or psychometrically tested, as their primary purpose was educational rather than evaluative.

In accordance with the flipped classroom logic, the preparatory phase was designed to shift the initial acquisition of foundational concepts to the pre-session moment, thereby allowing face-to-face time to focus on clinical application, collaborative reasoning, discussion, and argumentation^1–2,7−8^. At the beginning of the in-person activity, a brief review of the initial case and key concepts was conducted to facilitate participation among learners with different levels of prior preparation.

Consistent with contemporary CBCL recommendations, the preparatory materials were intentionally aligned with the educational objectives and organized as “advance organizers,” guiding learners regarding the essential concepts expected before the session and supporting self-regulated learning [1]. However, learner adherence to the preparatory materials was not formally measured, and the educational materials were not externally validated as independent instructional resources.

## Step 2: organization of the face-to-face session

The face-to-face session lasted a total of 90 min and was organized for residents divided into groups of four participants. The choice of this group size is consistent with the classic Harvard CBCL design, in which small groups of four students favored participation, individual accountability, and diversity of perspectives without generating excessive diffusion of responsibility [6].

The session schedule consisted of three linked stages organized around three sequential clinical cases. Case 1 (discitis-osteomyelitis), the same introductory case described in the case development section was used as an initial ten-minute guided review to activate prior knowledge and align participants’ clinical-radiological reasoning. This was followed by a forty-minute discussion of Case 2 (epidural abscess) and a forty-minute discussion of Case 3 (radiological mimic of spinal infection). Both Cases 2 and 3 followed a fixed three-stage CBCL structure. In the first stage, each resident prepared an individual response for five minutes. In the second stage, group members discussed their hypotheses and developed a consensus response over fifteen minutes. In the third stage, a twenty-minute plenary discussion was conducted, including comparison of group responses, faculty facilitation, and synthesis of key clinical and radiological concepts.

This structure aligns with the model described by Krupat et al., in which learners first respond individually to an open-ended question, then engage in small-group consensus building, and finally participate in a facilitated large-group discussion focused on refining reasoning and underlying principles [6–7].

## Stage 3: content domains and formative intentionality

The session was designed to integrate three educational domains explicitly indicated in the project slides. The first domain focused on basic concepts, especially anatomy, microbiology, and pathophysiology. The second domain focused on skills, that is, the incorporation of ACR Appropriateness Criteria into clinical practice. The third domain corresponded to the attitudinal domain, seeking to develop in residents the perception that the use of appropriateness criteria is important and necessary in clinical decision-making.

**Fig. 1:**
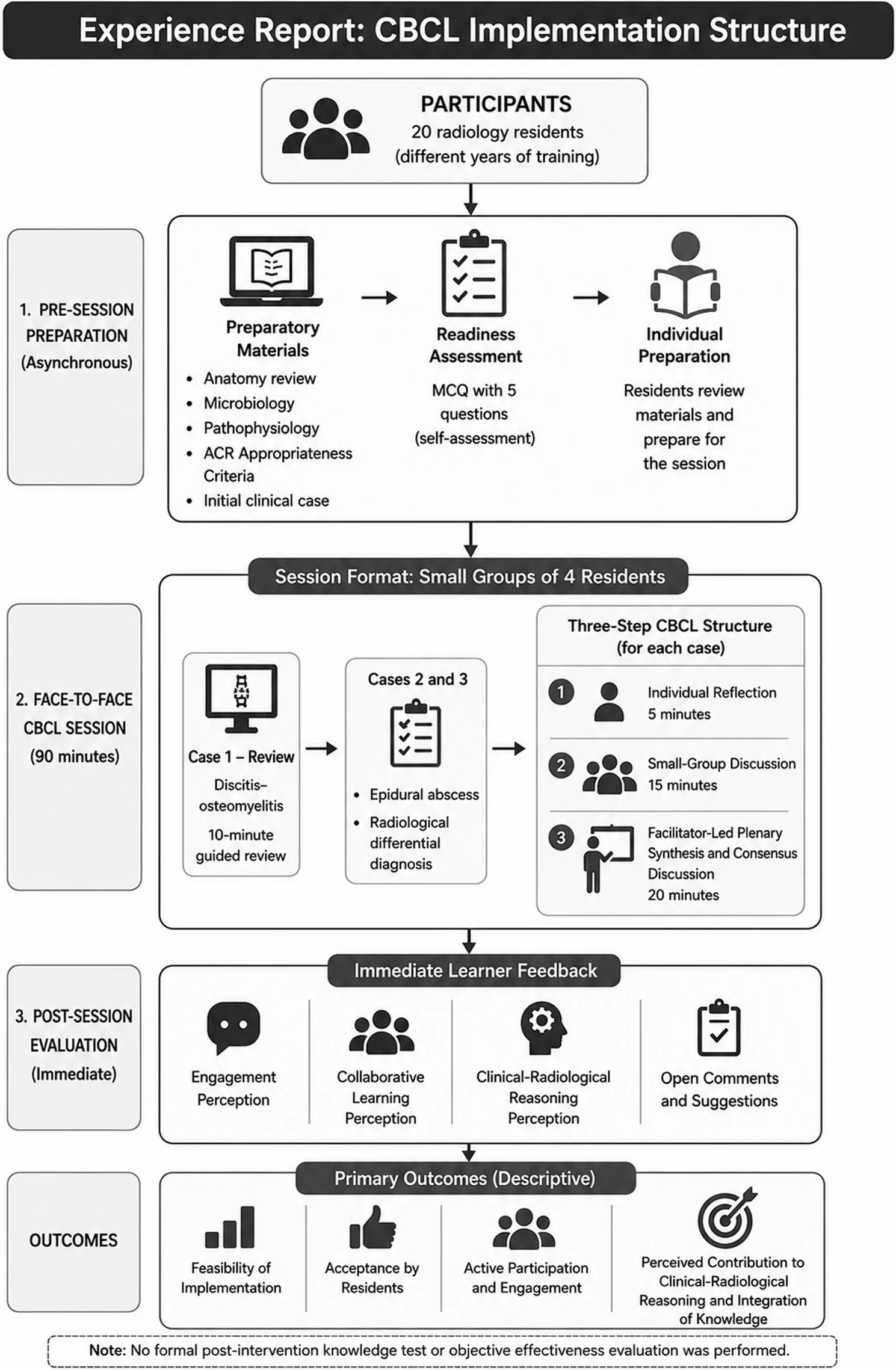
Demonstrates all stages of the CBCL process in a clearer and more didactic manner. Source: Authors 2026; image created using Canva

From a conceptual point of view, the anatomy section emphasized structures relevant to the spread and location of infection, such as the epidural space, the venous plexus, and the terminal arterioles of the vertebral plates. The microbiology section presented agents, sites of involvement, host factors, hematogenous routes, laboratory tests, and the main pathogens involved. Pathophysiology was addressed by articulating hematogenous dissemination, colonization in the terminal vertebral plate, extension to the disc, and spread to other compartments, including paravertebral tissues and the epidural space. This articulation between basic science and clinical application is consistent with one of the principles most emphasized in recent CBCL literature, which describes it as a strategy aimed at longitudinal and vertical integration between basic and clinical knowledge [1, 7–8]. Stages of the CBCL structure shown in Fig. 1.

### Case development

Case 1 (discitis-osteomyelitis), used as the introductory case of the session, presented a 55-year-old man with severe progressive neck pain, fever, painful limitation of cervical mobility, a recent history of aortic valve endocarditis, and altered inflammatory laboratory markers, including leukocytosis, elevated ESR (Erythrocyte Sedimentation Rate), and markedly increased CRP (C-Reactive Protein). The trigger question asked participants to indicate, based on anatomy, microbiology, and the American College of Radiology (ACR) criteria, which imaging tests they would order to confirm the suspected diagnosis and why. After formulating their hypotheses, participants were presented with T1-weighted, STIR (Short Tau Inversion Recovery), and T1-weighted MRI (Magnetic Resonance Imaging) findings with contrast and fat suppression, demonstrating changes in the C5–C6 vertebral endplates, consistent with vertebral infection in the clinical context provided.

The session then explicitly revisited key concepts, including nonspecific clinical presentation, pathophysiology, the importance of early diagnosis, the prognostic role of clinical suspicion, the appropriateness of MRI with or without contrast, and therapeutic principles.

This design allowed the case to go beyond the simple question of “which test to order,” functioning instead as a trigger for the clinical-radiological reasoning chain: diagnostic suspicion, clinical-laboratory correlation, selection of imaging modality, interpretation of findings, and understanding of the underlying pathophysiological mechanisms. This organization is consistent with Berkowitz and Chang’s recommendation that CBCL cases should be designed for the contextualized application of previously studied principles, rather than merely for content verification [2].

## Role of the facilitator

The facilitator did not assume the traditional lecturer role of lecturer. Instead, the facilitator guided the learning process by organizing the flow of the session, managing time, maintaining alignment with the learning objectives, and promoting productive comparison between individual responses and group consensus. Consistent with the CBCL model described by Krupat et al., the facilitator’s role was to help learners identify not only the most appropriate answer, but also the underlying clinical and conceptual principles supporting the reasoning process [6, 10].

The recent CBCL literature emphasizes that effective facilitation requires specific pedagogical preparation. Besche et al. describe successful facilitators as individuals capable of managing expert bias, calibrating case complexity, sustaining discussion without prematurely resolving uncertainty, and guiding learners toward conceptual synthesis [1]. In the present experience, facilitator mediation was therefore considered a central pedagogical component of the CBCL structure rather than merely organizational support [11].

To minimize passive participation and variability in group dynamics, the session followed a structured sequence consisting of individual reflection, small-group discussion, consensus building, and facilitator-led plenary synthesis. This organization aimed to preserve individual accountability while encouraging collaborative reasoning and active participation.

Facilitator performance was not formally assessed, and no standardized facilitator evaluation tool was used. Additionally, facilitator variability may have influenced learner engagement, depth of discussion, and conceptual synthesis, which is acknowledged as a limitation of the experience report. Although the model is potentially reproducible and scalable, successful implementation likely depends on adequate facilitator preparation, structured case design, protected instructional time, and institutional support.

## Evaluation of the experience

The evaluation of the intervention was designed on three levels: knowledge acquisition, immediate feedback from the learner, and subsequent change in attitudes or behaviors. To this end, knowledge assessment by multiple choice question (MCQ) was planned before and after the session, a feedback survey immediately after the session, and an audit of medical records six months later to be carried out by the residency program coordination.

Participant feedback was collected through a structured questionnaire with open-ended questions, administered immediately after the session. The instrument was designed to capture participants’ perceptions regarding engagement, development of clinical-radiological reasoning, collaborative learning, and perceived relevance to clinical practice. Data were analyzed using a qualitative descriptive approach, in which responses were reviewed and grouped according to recurring themes.

The local adaptation of this curriculum focused on immediate feedback from the learner. For feasibility reasons, the post-curriculum chart audit to assess behavioral change was not performed. A pre-session MCQ was administered for learner self-assessment; however, due to operational constraints, a post-session MCQ was not conducted. The immediate feedback showed a predominance of positive evaluations. Common elements identified included engagement, the contribution of group discussion to learning and clinical reasoning, the practical relevance of the case, and the intention to apply the “clinical + image + decision” reasoning approach in future practice. Four central themes emerged from the analysis: engagement, development of clinical reasoning, collaborative learning, and relevance to professional practice.

Our findings are consistent with prior CBCL studies reporting high learner engagement and perceived educational value [1, 3, 5–6]. However, similar to several previously published experiences, the present report relied predominantly on subjective learner perceptions rather than objective measures of reasoning performance or long-term behavioral change. This reflects an important limitation still present in a substantial portion of the CBCL literature and reinforces the need for future studies incorporating controlled designs and longitudinal educational outcomes [6].

## Discussion

Our experience suggests that CBCL may represent a pedagogically valuable approach when the educational objective extends beyond simple content transmission and seeks to support the organization of clinical reasoning in the context of complex diagnostic problems. In the setting of suspected spinal infection, the educational activity required learners to integrate multiple domains simultaneously, including clinical presentation, laboratory markers, anatomical foundations, microbiological dissemination pathways, imaging interpretation, and the application of appropriateness guidelines. This degree of cognitive integration appears particularly compatible with active learning approaches that emphasize hypothesis generation, collaborative reasoning, comparison of interpretations, and consensus building [5, 7].

An important aspect of the experience was the articulation between pre-session preparation and face-to-face discussion. By adopting a flipped classroom structure within the CBCL model, participants had prior exposure to essential concepts before the in-person activity, allowing classroom time to focus predominantly on clinical application, discussion, argumentation, and reasoning rather than passive exposition [1–2, 6]. Although learner adherence to preparatory materials was not formally measured, the introductory review conducted at the beginning of the session helped support participation among residents with different levels of prior preparation.

The sequence of individual responses followed by small-group consensus also appeared to contribute to active participation and learner accountability. This structure reduced the possibility of immediate dependence on more experienced peers and encouraged each participant to formulate an initial interpretation before collective discussion. Consistent with the CBCL model described in the Harvard educational literature, the process of contrasting different formulations and reasoning pathways functioned as an important pedagogical element for collaborative learning and conceptual refinement [6–7].

From a professional training perspective, the activity also sought to reinforce attitudes related to evidence-informed clinical decision-making. The incorporation of the American College of Radiology (ACR) Appropriateness Criteria [3] as an applied decision-support tool, not merely as theoretical content, helped contextualize the relationship between clinical judgment, radiological reasoning, and guideline-based practice. However, these contributions were inferred from the educational structure and participant perceptions rather than objectively measured outcomes.

As an educational experience report, this manuscript was not designed to evaluate educational effectiveness. Therefore, the findings should be interpreted as descriptive observations regarding implementation feasibility and participant perceptions rather than as evidence of learning gains, clinical competence, or behavioral change. Several limitations should be acknowledged in this regard. First, no objective measures of knowledge acquisition, clinical reasoning performance, or behavioral change were collected, as the primary purpose of the experience was to explore feasibility, implementation processes, and immediate learner perceptions. Although a readiness multiple-choice questionnaire (MCQ) was administered before the session, no post-session assessment or formal pre–post comparison was conducted due to operational and feasibility constraints. Likewise, a previously planned longitudinal chart audit could not be implemented within the local educational context. Consequently, the educational outcomes reported here should be interpreted with caution and viewed as preliminary observations that may inform future studies designed to evaluate effectiveness using objective outcome measures and longitudinal assessment strategies.

Second, the findings rely predominantly on subjective participant feedback summarized descriptively according to recurring themes, without systematic qualitative analysis, audio recording, or transcript-based evaluation. Therefore, claims regarding improvements in reasoning, engagement, or educational impact should be interpreted as perceived educational contributions rather than objectively demonstrated effects.

Third, the experience was conducted in a single institution, during a single educational activity, with a relatively small number of participants and without a control group, which limits external generalizability. Additionally, prior learner exposure to CBCL was not systematically measured, and participant motivation may have positively influenced the reception of the activity. Facilitator performance was also not formally evaluated, and variability in facilitation may influence learner engagement, depth of discussion, and conceptual synthesis.

The literature additionally highlights potential challenges associated with CBCL implementation, including perceived workload, variability in learner participation, need for calibrated case complexity, and the requirement for adequately prepared facilitators [1, 7]. These factors reinforce that successful implementation depends not only on case quality, but also on institutional support, protected instructional time, structured facilitation, and alignment between preparatory materials and learning objectives.

Despite these limitations, this experience report provides a detailed and contextually grounded description of CBCL adaptation and implementation within a Brazilian radiology residency program. The findings support the feasibility and acceptability of this pedagogical structure and suggest its potential value as an active learning strategy for promoting collaborative discussion and integration between foundational and clinical knowledge in postgraduate medical education [4, 8]. Future studies using objective educational outcomes, longitudinal follow-up, and controlled or multicenter designs are needed to further investigate the educational impact and reproducibility of this approach.

### Final considerations

This is one of the early attempts to implement CBCL in Brazilian radiology residency training. Our adaptation and implementation experience was feasible and well received by learners. The curriculum, focused on teaching clinical-radiological reasoning in suspected spinal infection, proved to be pedagogically consistent, theoretically grounded, and aligned with the available evidence on active learning. Anchored in prior preparation, small groups, open-ended questions, consensus building, and facilitator guidance, the educational intervention favored integration between anatomy, microbiology, pathophysiology, imaging, and clinical guidelines. More than simply a teaching technique, CBCL functioned as a pedagogical structure capable of reorganizing the role of the student, the teacher, and the clinical case in the teaching-learning process. Our experience offers concrete support for its further incorporation into residency programs and other medical education settings that require active learning, basic-clinical integration, and evidence-based decision-making.

## Data Availability

The datasets generated and/or analyzed during the current study are not publicly available because this study is an experience report based on educational practice and did not involve the collection of structured research data. No individual participant data were collected or analyzed. This study does not constitute human subjects research according to Brazilian regulations (Resolution CNS No. 510/2016). Additional information about the educational materials used in this study is available from the corresponding author upon reasonable request.
